# A flipped classroom improves medical students’ skills how to manage medical emergencies—an intervention-control study

**DOI:** 10.1186/s12909-025-07898-x

**Published:** 2025-10-07

**Authors:** M. Ruesseler, V. Britz, A. Herrmann-Werner, T. Festl-Wietek, C. Weckwerth, L. Bepler, Y. Beaugé, J. Sterz

**Affiliations:** 1 Goethe University, Institute for Medical Education and Clinical Simulation, Director Prof. M. Ruesseler, Goethe University, Theodor Stern Kai 7, 60590 Frankfurt, Germany; 2 Tübingen Institute for Medical Education, University of Tuebingen, Tuebingen, Germany; 3 Faculty of Psychology, Fern Universität Hagen, Universitätsstr. 37, 58097 Hagen, Germany

**Keywords:** Flipped classroom, Emergency medicine, Simulation training, Undergraduate medical education

## Abstract

**Background:**

Emergency medical skills must be mastered by every doctor. Early implementation of these skills in medical education is, therefore, essential. A form of realization is simulation-based training, which offers several advantages: it can be implemented at low threshold in undergraduate medical curricula, induces comparable stress levels to doctors in real-life situations and improves patient morbidity and mortality.

Additionally, knowledge acquisition can be improved via e-learning. Teaching formats such as flipped classrooms have proven to be effective. However, it remains unclear whether the flipped classroom is also more effective than traditional classroom teaching in preparing students to apply emergency medicine competencies in realistic simulation scenarios.

Therefore, this study aims to analyze the influence of an e-learning based flipped classroom approach combined with a simulation-based training on the acquisition of competencies in practical emergency measures compared to a classic classroom plus simulation course.

**Methods:**

This prospective intervention-control study is based on a standardized, simulation-based training designed to increase undergraduate medical students’ skills in managing medical emergencies. Participants were assigned to one of two groups: the control group received classic classroom-based theoretical input prior to simulation training, while the study group followed a flipped classroom approach, completing the same theoretical content through e-learning modules. Duration of theoretical input and simulation-based training was identical for both groups. Outcomes were assessed using a formative OSCE, consisting of six emergency case scenarios, based on standardized checklists.

**Results:**

165 students participated in the study (86 Control group, 79 study group). Participants in the flipped classroom training group scored significantly higher in all six emergency case scenarios.

**Conclusion:**

This study shows that an e-learning-based flipped classroom approach, when combined with simulation training and supported by well-designed instructional content, can improve the acquisition of practical emergency medicine skills compared to a traditional classroom format with identical simulation training.

**Supplementary Information:**

The online version contains supplementary material available at 10.1186/s12909-025-07898-x.

## Background

Emergency medical skills must be mastered by every doctor regardless of their area of practice. This is particularly important in terms of patient outcomes in emergency situations. Polytraumatized patients in particular benefit from guideline-based treatment based on standardized algorithms [1, 2]. Especially when treating emergency patients, the combination of solid factual and application knowledge is indispensable, as time-critical decisions often have to be made here. However, especially young physicians at the beginning of their career are confronted with the fact that their current level of training is not sufficient to master their daily work and in particular emergency situations adequately [3–5].

With this in mind, the early implementation of emergency procedures and treatment of emergency patients in medical education beginning from the undergraduate study term onwards seems necessary. For this, simulation-based training offers a number of advantages: Studies have shown that the incorporation of in situ simulation trainings improve patient morbidity and mortality [6, 7]. Moreover, simulated emergency scenarios are able to induce stress levels that are comparable to the stress levels which medical doctors experience in real-life situations [8]. Simulation-based training is also proven to be an easy implementable teaching method for training undergraduate medical students in curricular emergency medicine courses [9].

Various studies have shown that short-term and long-term acquisition of knowledge can be improved via e-learning [10–12]. Teaching formats such as the flipped classroom approach have proven to be particularly effective [13, 14]. Flipped classroom is defined as a blended-learning method with a self-learning phase before classroom teaching [15]. As a result, factual and application knowledge, which is located at a low taxonomy level according to Bloom [16], can be acquired in asynchronous teaching formats before applying and deepening acquired knowledge in the synchronous classroom phase [15]. The flipped classroom approach is highly appreciated by learners [17–19].

Despite numerous studies and meta-analyses demonstrating the positive effects of flipped classrooms in health professions education, most research has focused on knowledge acquisition [18, 20] rather than its transfer to practical skill application [19]. For instance, Zheng et al. demonstrated that using a flipped classroom approach for training medical undergraduate students in mass casualty triage improved outcomes in knowledge-based multiple-choice tests compared to traditional lecture-based instruction [18]. However, this improvement did not translate into better performance during a simulated field triage [18], highlighting shortcomings in fostering practical skill application. Similarly, Beom et al. found no statistically significant advantage of flipped-classroom training over traditional methods in advanced cardiopulmonary life support during a simulated emergency situation [21].

Recent studies have reported more promising outcomes for flipped classroom approaches in emergency medicine, such as improved cardiopulmonary resuscitation skills in nursing students [22], enhanced team performance during advanced life support [23], and improved skills acquisition in newborn resuscitation scenarios [24]. However, these studies often focus on isolated competencies, involve small sample sizes, or examine only one aspect of emergency care. As a result, evidence for the broader effectiveness of flipped classroom models remains limited.

This study addresses that gap by evaluating an e-learning-based flipped classroom combined with simulation training. By assessing the acquisition of competencies across diverse emergency scenarios in a larger cohort, we aim to provide a more comprehensive and rigorous evaluation of its impact on practical skills acquisition in undergraduate medical education.

## Methods

### Study design

This prospective study had an intervention-control design based on a standardized, simulation-based training designed to increase undergraduate medical students’ skills in managing medical emergencies. In this context, 'skills' refer to an integrated set of competencies, including structured patient assessment clinical reasoning, procedural execution, and team-based communication, all of which are essential in managing acute emergency scenarios within OSCEs.

### Study participants

Study participants were undergraduate medical students in their 5th year of a six-year program at the medical faculty of Goethe University, Frankfurt, Germany. The study was Conducted between October 2020 and July 2021.

All students were informed of the study content prior to their training. Participation in the study was voluntary and took place after written informed consent had been given, which was revocable at any time. All invited students agreed to participate in the study and provided written informed consent. Basic data regarding student age and gender were collected using an online questionnaire.

The Ethics Committee of the Faculty of Medicine, Goethe University Frankfurt, confirmed that no formal ethics approval was required for this type of educational research, as it does not constitute a biomedical research project in the sense of the Declaration of Helsinki and the requirements of §15 of the Professional Code of Conduct for Physicians in Hesse. The study was conducted in accordance with the Declaration of Helsinki.

### Study protocol

Students were completing their obligatory training in emergency medicine ( ). This consists of a one-day training of medical first aid in year 1 or 2, lectures in year 3 and 4 followed by a written multiple-choice test. The training is completed by a training week in the 5th year, within which the present study was conducted. Students were assigned to two groups: an intervention group and a control group (Table 1). The assignment to these groups was carried out by the Student Administration Office of Goethe University. Neither the authors nor participation in the study influenced this assignment.

**Table 1:** Course design with modules and learning objectives for classic and flipped classroom

Modul	Objectives	Execution	
		**Classic Classroom**	**Flipped Classroom**
Basic Competencies	◦ Basic life support ◦ Airway Management ◦ Monitoring ◦ i.v.-Access and drug administration	4 × 90 min theory + hands-on (Day 1)	4 × 45 min e-learning (Day 0) 4 × 45 min hands-on (day 1)
Emergency Management	◦ Cardiopulmonary resuscitation Cardinal Symptom: ◦ Chest pain ◦ Dyspnea ◦ Impaired consciousness ◦ Cardiopulmonary resuscitation repetition and ROSC	4 × 120 min theory + hands-on each (Day 2/3) 60 min theory + hands-on (Day 3)	4 × 60 min e-learning (Day 2) 4 × 60 min hands-on (Day 3) 15 min e-learning (Day 2) 45 min hands-on (Day 3)
Formative OSCE	◦ Bystander CPR with AED ◦ Acute asthma ◦ Stroke ◦ Myocardial infarction ◦ ACLS Ventricular fibrillation ◦ Sepsis	6 ECOS à 8 min + 4 min Feedback (Day 4)	

#### Control group

In this training week, on day one, students repeat and expand their basic competencies in managing medical emergencies rotating in small groups (max. 6 students per group) through four modules (90 min per module) (Table 1): Basic life support, monitoring and defibrillation, iv-access and drug administration, and airway management. Each module is highly standardized with a structured, detailed blueprint and consists of theoretical inputs and hands-on sessions.

On day two and three, students rotate through four different modules with 120 min per module (‘cardio-pulmonary resuscitation’, cardinal symptoms: ‘chest pain’, ‘dyspnea’, and ‘impaired consciousness’). In ‘cardio-pulmonary resuscitation’, students develop, under the guidance of the instructor, the algorithm of advanced cardiac life support as well as the different assignments within the team. The major part of the module consists of practical training in resuscitation skills on simulated in-house cardiac arrest scenarios. A simulation mannequin (Resusci Anne, Laerdal, Stanvanger, Norway) is used for the hands-on training.

In the theoretical parts within the three cardinal symptoms modules, students develop under structured guidance of the instructor a mind map of the typical ‘ABCDE’- and ‘SAMPLER’-results (ABCDE – Airway, Breathing, Circulation, Disability, Environment; SAMPLER – Symptoms, Allergies, Medication, Past medical history, Last oral intake, Events, Risk factors) and the possible differential diagnoses and discuss their required measures e.g. drug administration. The hands-on consists of two scenarios for each of the three cardinal symptoms. For the scenario’s, simulated patients are deployed. During the hands-on sessions, all participants are active for the entire duration of scenarios rotating through the different team member roles. Each scenario starts with a standardized briefing and concludes with structured feedback highlighting three to four important issues that were good and should be maintained and issues that could be improved and/or changed in the next scenario.

The two days end for all students with a second module of ‘cardiopulmonary resuscitation’ (60 min) as repetition for the resuscitation skills as well as to deepen the theoretical and practical aspects of ROSC (recovery of spontaneous circulation).

### Study group

For the study group, the flipped class room design was grounded in the community of inquiry framework (COI), incorporating cognitive presence through e-learning, social presence via team-based simulations, and teaching presence through instructor facilitation and structured feedback [25, 26]. To implement this approach, all theoretical input was converted into e-learning modules, which included specific learning activators such as videos, quizzes, and case-based exercises with accompanying images. These modules were carefully designed to align with the learning objectives and instructional methods originally outlined in the detailed training blueprint, ensuring consistency with the overall curriculum. The time for each e-learning module was held equal to the classic classroom training (Table 1). The e-learning modules were delivered using the ‘Lernbar’ platform, a digital tool developed by the University of Frankfurt, Germany. This platform allows for the creation, customization, and distribution of learning content to specific user (https://lernbar.uni-frankfurt.de).

The training of basic competencies took place during day one and two. Whereof, students had to complete four e-learning modules on day one and the corresponding hands-on modules on day two. The e-learning component was designed to be asynchronous and flexible, allowing students to complete it at their own pace. All students accessed the e-learning materials on their private laptops, typically from home or another self-selected environment.

On day three, students completed the e-Learning of the module’s cardio-pulmonary resuscitation, the cardinal symptoms and ROSC including the theoretical inputs of ABCDE, SAMPLER, differential diagnosis and team members. On day four, the hands-on training with the scenarios took place. The scenarios, briefing and feedback were equal in content and duration to the regular training.

### Instructors

For both groups, instructors were physicians, paramedics and peer students. In each training module, two instructors were present, one of which being the main instructor for the theoretical and practical parts, including briefing and feedback. The second instructor was in the background organizing the unobstructed sequence of the scenarios (control of mannequin/support for simulated patients, equipment, function of monitor) and gave short further inputs in the theoretical parts and the feedback.

Before becoming instructors, applicants had to complete the training week as participant and received a detailed blueprint and manual for each training module. They completed a detailed didactic training regarding teaching methods, presenting methods, briefing and debriefing. Afterwards, they participated as second instructor and slowly took over the role of the first instructor under supervision. Furthermore, all instructors are supervised regularly.

### Measurement

For both groups, the measurement took place on the last day of the training week as part of the regular training. Here, students complete a formative objective structured clinical examination (OSCE) consisting of six emergency case OSCE station (ECOS) [9]. The ECOS cover the disease pattern of the previous training (Table 1) supplemented by the clinical picture of sepsis, which was not part of the prior learning objectives during the previous days. Students had 8 min per station plus 4 min for receiving feedback. In each ECOS, students should demonstrate their competencies learned during the training week. In contrast to the training week where students were in a team of maximum 6 students being either the team leader or a team member, in the ECOS, each student had to manage each emergency as the team leader accompanied by a trained tutor playing the role of the assistant following the instructions of the team leader. The patients were either trained SPs (acute asthma, stroke, myocardial infarction, sepsis) or mannequins (Bystander CPR, Ventricular fibrillation).

Each ECOS was rated on a detailed checklist which was task-specific but had an identical structure based on the ABCE algorithm taught in the training and comprised rigorous step-by-step procedures (see Supplementary File 1 for the full checklists). The examiners were physicians, paramedics and peer students and trained using the checklists as well as in providing structured feedback.

### Data analysis

An a-priori sample size analysis (two-sided; set for: Mean differences for two independent means (two groups)) was performed using G*power [27]. Based on an estimated effect size of 0.6 in the allocation ratio of 1 (N2/N1), using an alpha error prob. of 0.05 and a power of 0.95, a sample size of 74 per group was estimated. The effect size of 0.6 was informed by prior studies, including Rui et al. [28] and Beom et al. [21]. While Beom et al. used an effect size of 0.68, derived from Rui et al., their study observed only a near-significant trend favoring the flipped classroom model for the overall score. To account for this trend and adopt a more conservative estimation, we adjusted the effect size to 0.6, ensuring adequate power to detect a moderate effect in our study. Data was normally distributed. Means, standard deviations, frequencies and percentages were calculated. In order to test for possible differences between intervention and control group, t-test for independent samples were performed. The data analysis was performed by SPSS Statistics version 27 (IBM, Armonk, NY). The level of *p* < 0.05 was reported as significant.

## Results

A total of 165 students participated in the present study. 86 students completed the regular training, 79 students were in the study group. Table 2 shows the demographic characteristics of the students.

**Table 2:** Demographic characteristics of the students

	Total	Flipped classroom	Classic Classroom
Participants N (Female)	165 (108)	79 (50)	86 (58)
Age (years)	25.4	25.4	25.3

Data presented as absolute numbers or mean ± standard deviation

**Fig. 1:**
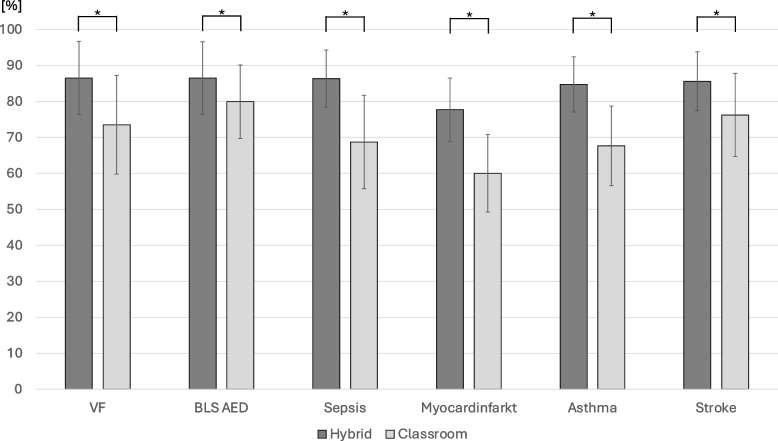
Data are presented as mean (percentage of total possible points) + standard deviation flipped classroom (dark grey) versus classic classroom (light grey)

The results of the t-test for independent groups showed, that participants of the flipped classroom training scored significantly (*p* < 0.001) higher in the total score at all six Emergency Case OSCE stations (Fig. 1).

The items of each station were clustered to item groups to improve comparability of the different stations. E.g. the item group ‘basic measures’ includes assessment of scene/safety/situation, approaching the patient, embedding of team members, or optimizing setting. Here too, the participants in the flipped classroom training showed significantly better results in all but two clusters. Table 3 shows the results of the item groups.

**Table 3:** Results of all six formative Emergency case OSCE stations presented in total as well as for major items groups

	**Training approach**	**Total (%)**	**Basic measures (%)**	**ABCDE (%)**	**Patient history (%)**	**Management (%)**
		Mean ± SD	Mean ± SD	Mean ± SD	Mean ± SD	Mean ± SD
**Ventricular fibrillation**	Flipped	86.5 ± 10.2	91.5 ± 15.2	89.1 ± 9.3	-	85.3 ± 17.2
	Classic	73.5 ± 13.7	84.4 ± 17.2	78.3 ± 13.2		66.7 ± 24.2
	***p*-value**	<.01	<.01	<.001		<.001
**BLS AED**	Flipped	86.5 ± 10.1	96.0 ± 8.1	89.7 ± 10.3	-	86.7 ± 17.0
	Classic	79.9 ± 10.2	88.4 ± 15.4	87.3 ± 8.4		71.9 ± 23.0
	***p*-value**	<.001	<.001	n.s		<.001
**Sepsis**	Flipped	86.3 ± 7.9	89.7 ± 17.9	87.3 ± 8.8	82.4 ± 12.0	86.7 ± 15.7
	Classic	68.7 ± 13.0	81.2 ± 14.0	73.4 ± 12.3	73.5 ± 12.3	66.2 ± 19.6
	***p*-value**	<.001	<.001	<.001	<.001	<.001
**Myocardial infarction**	Flipped	77.7 ± 8.8	86.7 ± 13.5	79.6 ± 10.5	71.8 ± 13.7	90.5 ± 12.6
	Classic	60.0 ± 10.8	65.1 ± 12.7	55.0 ± 9.7	49.1 ± 22.5	79.3 ± 14.8
	***p*-value**	<.001	<.001	<.001	<.001	<.001
**Asthma**	Flipped	84.7 ± 7.7	92.9 ± 10.7	85.1 ± 9.8	81.7 ± 14.1	89.2 ± 10.4
	Classic	67.6 ± 11.1	77.0 ± 13.4	65.2 ± 12.0	69.7 ± 25.3	71.1 ± 14.0
	***p*-value**	<.001	<.001	<.001	<.001	<.001
**Stroke**	Flipped	85.6 ± 8.2	90.0 ± 14.3	88.5 ± 11.2	95.1 ± 10.5	85.6 ± 12.1
	Classic	76.2 ± 11.5	98.0 ± 7.6	78.1 ± 14.7	70.8 ± 21.0	87.9 ± 14.2
	***p*-value**	<.001	<.001	<.001	<.001	n.s

Data are presented as mean (percentage of total possible points) ± standard deviation

(*BLS* Basic Life Support, *AED* automated external defibrillator, *ABCDE* Airway, Breathing, Circulation, Disability, Environment). *n.s.* not significant

## Discussion

This study demonstrates that a flipped classroom approach, when combined with simulation-based training can more effectively foster the acquisition of practical skills in emergency medicine than a traditional classroom-based format with identical simulation exposure. This is reflected in the significantly higher OSCE scores observed in our study group compared to participants in the traditional classroom-based course. While prior research has largely focused on the benefits of flipped classrooms for theoretical knowledge acquisition [18, 20, 29–31], our findings suggest that such an approach, when instructionally sound and aligned with practical learning objectives, also enhances practical skills. To support the interpretation of the observed OSCE scores, we refer to Kane’s framework of assessment validity [32], which conceptualizes a chain of inferences linking observed performance to broader claims about ability. In our study, structured OSCE stations reflected authentic emergency scenarios and assessed practical skills such as patient assessment, clinical reasoning, procedural execution, and team-based communication. In line with Kane’s framework, the use of trained raters, a standardized checklist, and realistic simulation tasks strengthens the validity argument by supporting the scoring, generalization, and extrapolation inferences [32]. This justifies a cautious interpretation of the observed score differences as indicative of improved practical skills. Although the flipped classroom primarily targets cognitive preparation, it may indirectly support the execution of manual or procedural tasks. By engaging with structured digital content beforehand, learners are likely to form clearer procedural schemata and reduce cognitive load during hands-on training [35] This cognitive readiness can enhance the quality of psychomotor performance in simulation, particularly under time pressure [35]. Such effects are supported by cognitive load theory [33, 34], which posits that well-organized prior knowledge enables more effective task execution in complex, simulation-based environments [33–36].

These findings are consistent with prior research showing benefits of flipped classroom models when implemented with high-quality instructional components. For example, Ge et al. (2020), Conducted a meta-analysis of 19 studies involving 2114 participants highlighted the superior impact of flipped classrooms on theoretical knowledge and practical skills compared to traditional lectures in radiology education [29]. Similarly, Hu et al. (2019) demonstrated that combining flipped classrooms with problem-based learning leads to better outcomes in knowledge acquisition and application compared to traditional lecture-based teaching [30].

However, not all studies have reported positive outcomes. Beom et al. (2018) and Zheng et al. (2022) found no statistically significant advantage of flipped classroom methods over traditional teaching in advanced cardiopulmonary life support [21] and mass casualty triage scenarios [18], respectively. Beom et al. highlighted key limitations, including the short duration of the flipped classroom intervention (1 h), potential cross-contamination between groups, and challenges in ensuring adequate student preparation [21]. They also noted that their small sample size (*n* = 108) limited statistical power, despite observing trends favoring the flipped classroom group [21]. Zheng et al. similarly attributed their results to limited sample size (*n* = 103) and suggested the need for larger studies to improve statistical power [18]. By contrast, our study, with a larger sample size (*n* = 165) and carefully designed intervention, addresses most of these limitations and provides robust evidence for the efficacy of flipped classrooms in enhancing procedural skills.

### The design of the e-learning based flipped classroom module

A key factor influencing the success of flipped classrooms is the design of the e-learning component, particularly in fostering cognitive presence as defined by the COI framework. Cognitive presence involves creating opportunities for learners to construct meaning through interaction with the content [25, 26, 37]. For instance, Chang et al. demonstrated that students engaging with multimedia materials (e.g., videos, photos, and text) outperformed those provided only with traditional textbooks in ECG interpretation during an ACLS course [38]. By contrast, Beom et al. used PowerPoint files with recorded explanations as pre-class material and found no significant advantage of the flipped classroom approach [21].

In our study, the e-learning modules incorporated interactive elements such as videos, quizzes, and case-based scenarios, all designed to promote learner autonomy and critical engagement. This intentional focus on cognitive presence likely enabled students to approach in-class simulation training better prepared, contributing to their superior performance in OSCEs.

### Team-based simulation training

Social presence, the second pillar of the COI framework, emphasizes collaborative learning and interaction among participants [25, 26, 37]. This concept is especially critical in emergency medicine, where translating theoretical knowledge into practical application, combined with effective teamwork and clear communication, is vital for providing timely and accurate patient care in high-pressure, team-based simulation scenarios.

Both the flipped classroom and traditional classroom groups participated in identical simulation-based training sessions, which provided hands-on opportunities to practice critical team-based skills such as role allocation, effective communication, and adherence to emergency protocols. However, the flipped classroom approach showed better results than the participants in the control group, even in the major item groups, involved core practical elements such as the correct implementation of the ABCDE algorithm. By engaging students in self-directed, interactive e-learning modules prior to these sessions, the flipped classroom approach may have enhanced students’ readiness to engage actively and confidently in the collaborative simulation environment [39]. Evidence from similar studies reinforces this interpretation. Hassan et al. demonstrated, that students who have been taught using a simulation-based flipped classroom performed significantly better during a simulated CPR than the ones being taught using a traditional simulation training [22]. This aligns with the findings of Moll-Khosrawi et al., who showed that the use of a simulation-based flipped learning leads to superior non-technical skills compared to a conventional lecture-based approach combined with simulation [40]. Beyond that, Hu et al. demonstrated that the implementation of flipped classroom combined with problem-based learning made students feel more improvement in abilities like communication, clinical thinking and teamwork [30].

### The instructor’s role

The role of the instructor, the third pillar of the COI framework, is pivotal in flipped classrooms, particularly during face-to-face interactions [25, 26, 37]. In successful flipped classroom formats, students perceive the lecturer more as a moderator who promotes interaction and exchange between students and is available to help with questions [41]. Furthermore, a recent study by Zheng et al. showed that in a curriculum that incorporates flipped classrooms, the use of peer-learning strategies (e.g., working with classmates to complete assignments or discussing course materials with a group of other students) and help seeking behavior are positive predictors of learning success [42]. These two elements were integral to our flipped classroom design. In addition, from the learners' perspective, it is not only important that the pre-class material is of high quality, but also that sufficient feedback is integrated [43]. Feedback proves efficacious by enabling students to compare their current level of knowledge with the desired target state [44]. Therefore, when developing the e-learning used in the flipped classroom during the present study, conscious attention was paid to combining different teaching formats such as texts, videos or case-presentations and regular questions to test knowledge with direct feedback for the learners. In contrast to this, in classic classroom teaching, interactive lessons were led by the instructor, but the individual learning pace or combination of different learning activators was not taken into account. This may be another reason for the superiority of the flipped classroom format compared to classic classroom teaching in the present study.

### Limitations

This study has limitations that should be acknowledged. First, the study was conducted at a single institution within a specific medical discipline, which may limit the generalizability of the findings. Although the groups were comparable in age and gender, the lack of data on prior academic performance introduces potential bias. Future studies should control for baseline academic differences to ensure a more comprehensive evaluation of flipped classroom outcomes. Another possible explanation for the superiority of students in the intervention group may be that they are more motivated than in other teaching formats [45] and have invested more time in preparation [30]. However, the present study did not analyze how much time the students actually invested in preparing during the e-learning. Subsequent studies should therefore take this into account. Furthermore, future research could also consider the temporal gap between accessing e-learning materials and applying the acquired knowledge. The temporal proximity between theory and practice was kept relatively short, potentially resulting in a priming effect (first introduced by Meyer & Schvaneveldt, 1971 [46]), which, while generally advantageous in facilitating information processing, retrieval, and enhancing learning performance, may overlook crucial information that could have significant adverse consequences in emergency situations. A greater temporal distance might attenuate a potential priming effect.

## Conclusion

A flipped classroom format, when supported by well-structured, didactically sound e-learning content and combined with simulation training, has the potential to improve practical competencies in emergency medicine compared to a traditional classroom course.

## Supplementary Information

Supplementary Material 1.

## Data Availability

The datasets used and/or analysed during the current study are available from the corresponding author upon reasonable request.

## Abbreviations

OSCE: Objective structured clinical examination
ECOS: Emergency case OSCE station

## References

[1] van Olden GDJ, Meeuwis JD, Bolhuis HW, Boxma H, Goris RJA. Advanced trauma life support study: trauma resuscitation time. Eur J Trauma. 2003;29:379–84.

[2] Teuben M, Löhr N, Jensen KO, et al. Improved pre-hospital care efficiency due to the implementation of pre-hospital trauma life support (PHTLS®) algorithms. Eur J Trauma Emerg Surg. 2020;46:1321–5. 31079191 10.1007/s00068-019-01141-1

[3] Ochsmann EB, Zier U, Drexler H, Schmid K. Well prepared for work? Junior doctors’ self-assessment after medical education. BMC Med Educ. 2011;11:99. 22114989 10.1186/1472-6920-11-99 PMC3267657

[4] Stefanescu M-C, Sterz J, Hoefer SH, Ruesseler M. Young surgeons’ challenges at the start of their clinical residency: a semi-qualitative study. Innov Surgical Sci. 2018;3:235–43. 10.1515/iss-2018-0015 PMC6604589 31579787

[5] Illing JC, Morrow GM, Rothwell nee Kergon CR, et al. Perceptions of UK medical graduates’ preparedness for practice: a multi-centre qualitative study reflecting the importance of learning on the job. BMC Med Educ. 2013;13(1):1–12. 10.1186/1472-6920-13-34 PMC3599362 23446055

[6] Goldshtein D, Krensky C, Doshi S, Perelman VS. In situ simulation and its effects on patient outcomes: a systematic review. BMJ Simul Technol Enhanc Learn. 2020;6:3–9. 10.1136/bmjstel-2018-000387 PMC8936935 35514456

[7] Johansson J, Blomberg H, Svennblad B, et al. Prehospital trauma life support (PHTLS) training of ambulance caregivers and impact on survival of trauma victims. Resuscitation. 2012;83:1259–64. 22366502 10.1016/j.resuscitation.2012.02.018

[8] Dias RD, Neto AS. Stress levels during emergency care: a comparison between reality and simulated scenarios. J Crit Care. 2016;33:8–13. 26987261 10.1016/j.jcrc.2016.02.010

[9] Ruesseler M, Weinlich M, Müller MP, Byhahn C, Marzi I, Walcher F. Simulation training improves ability to manage medical emergencies. Emerg Med J. 2010;27:734–8. 20852280 10.1136/emj.2009.074518

[10] Gaupp R, Körner M, Fabry G. Effects of a case-based interactive e-learning course on knowledge and attitudes about patient safety: a quasi-experimental study with third-year medical students. BMC Med Educ. 2016;16:1–8. 27400872 10.1186/s12909-016-0691-4 PMC4940690

[11] Pei L, Wu H. Does online learning work better than offline learning in undergraduate medical education? A systematic review and meta-analysis. Med Educ Online. 2019;24:1666538. 31526248 10.1080/10872981.2019.1666538 PMC6758693

[12] Co M, Cheung KYC, Cheung WS, et al. Distance education for anatomy and surgical training–a systematic review. Surgeon. 2022;20:95–205. 10.1016/j.surge.2021.08.001 PMC8514899 34483055

[13] Hew KF, Lo CK. Flipped classroom improves student learning in health professions education: a meta-analysis. BMC Med Educ. 2018;18:1–12. 29544495 10.1186/s12909-018-1144-z PMC5855972

[14] Graham KL, Cohen A, Reynolds EE, Huang GC. Effect of a flipped classroom on knowledge acquisition and retention in an internal medicine residency program. J Grad Med Educ. 2019;11:92–7. 30805104 10.4300/JGME-D-18-00536.1 PMC6375314

[15] Tolks D, Schäfer C, Raupach T, et al. An introduction to the inverted/flipped classroom model in education and advanced training in medicine and in the healthcare professions. GMS J Med Educ. 2016;33:46. 10.3205/zma001045 PMC4894356 27275511

[16] Bloom BS. A taxonomy for learning, teaching, and assessing: A revision of Bloom's taxonomy of educational objectives. Longman; 2010.

[17] Tan E, Brainard A, Larkin GL. Acceptability of the flipped classroom approach for in-house teaching in emergency medicine. Emerg Med Australas. 2015;27:453–9. 26235786 10.1111/1742-6723.12454

[18] Zheng Z, Yuan S, Huang M, et al. Flipped classroom approach used in the training of mass casualty triage for medical undergraduate students. Disaster Med Public Health Prep. 2022;16:94–101. 32762784 10.1017/dmp.2020.162

[19] Chen F, Lui AM, Martinelli SM. A systematic review of the effectiveness of flipped classrooms in medical education. Med Educ. 2017;51:585–97. 28488303 10.1111/medu.13272

[20] Uther P, Van Munster KA, Briggs N, O’Neill S, Kennedy S. Introducing early-phase medical students to clinical paediatrics using simulation and a flipped-classroom. J Paediatr Child Health. 2019;55:1107–12. 30672066 10.1111/jpc.14366

[21] Beom JH, Kim JH, Chung HS, Kim SM, Ko DR, Cho J. Flipped-classroom training in advanced cardiopulmonary life support. PLoS ONE. 2018;13:e0203114. 30183739 10.1371/journal.pone.0203114 PMC6124753

[22] Hassan EA, Elsaman SEA. The effect of simulation-based flipped classroom on acquisition of cardiopulmonary resuscitation skills: a simulation-based randomized trial. Nurs Crit Care. 2023;28:344–52. 35801367 10.1111/nicc.12816

[23] Ohlenburg H, Arnemann PH, Hessler M, Görlich D, Zarbock A, Friederichs H. Flipped classroom: improved team performance during resuscitation training through interactive pre-course content - a cluster-randomised controlled study. BMC Med Educ. 2024;24(1):459. 10.1186/s12909-024-05438-7. 38671434 10.1186/s12909-024-05438-7 PMC11046966

[24] Abou-Zamzam A, McCaw J, Niarison HR, Ravelojaona VA, Shilkofski N. Cross-sectional study in Madagascar demonstrates efficacy of virtual mentoring and flipped classroom modifications of neonatal resuscitation programme helping babies breathe. Acta Paediatr. 2023;112(8):1783–9. 10.1111/apa.16819. 37159532 10.1111/apa.16819

[25] Garrison DR, Arbaugh JB. Researching the community of inquiry framework: review, issues, and future directions. Internet High Educ. 2007;10(3):157–72.

[26] Garrison DR, Anderson T, Archer W. The first decade of the community of inquiry framework: a retrospective. Internet High Educ. 2010;13(1–2):5–9.

[27] Faul F, Erdfelder E, Lang A-G, Buchner A. G* Power 3: A flexible statistical power analysis program for the social, behavioral, and biomedical sciences. Behav Res Methods. 2007;39:175–91. 17695343 10.3758/bf03193146

[28] Rui Z, Lian-rui X, Rong-zheng Y, et al. Friend or foe? Flipped classroom for undergraduate electrocardiogram learning: a randomized controlled study. BMC Med Educ. 2017;17:53. 10.1186/s12909-017-0881-8. 28270204 10.1186/s12909-017-0881-8 PMC5341445

[29] Ge L, Chen Y, Yan C, Chen Z, Liu J. Effectiveness of flipped classroom vs traditional lectures in radiology education: a meta-analysis. Medicine (Baltimore). 2020;99:e22430. 33019421 10.1097/MD.0000000000022430 PMC7535556

[30] Hu X, Zhang H, Song Y, et al. Implementation of flipped classroom combined with problem-based learning: an approach to promote learning about hyperthyroidism in the endocrinology internship. BMC Med Educ. 2019;19:290. 31362729 10.1186/s12909-019-1714-8 PMC6668058

[31] Ke L, Xu L, Sun L, et al. The effect of blended task-oriented flipped classroom on the core competencies of undergraduate nursing students: a quasi-experimental study. BMC Nurs. 2023;22:1. 36624445 10.1186/s12912-022-01080-0 PMC9830926

[32] Kane MT. Validating the interpretations and uses of test scores. J Educ Meas. 2013;50:1–73. 10.1111/jedm.12000.

[33] Sweller J. Cognitive load during problem solving: effects on learning. Cogn Sci. 1988;12(2):257–85.

[34] Sweller J, Ayres P, Kalyuga S. Cognitive load theory. New York: Springer; 2011.

[35] Fraser KL, Ayres P, Sweller J. Cognitive load theory for the design of medical simulations. Simul Healthc. 2015;10(5):295–307. 10.1097/SIH.0000000000000097. 26154251 10.1097/SIH.0000000000000097

[36] Paas F, Renkl A, Sweller J. Cognitive load theory and instructional design: recent developments. Educ Psychol. 2003;38(1):1–4. 10.1207/S15326985EP3801_1.

[37] Lee YH, Kim K-J. Enhancement of student perceptions of learner-centeredness and community of inquiry in flipped classrooms. BMC Med Educ. 2018;18:1–6. 30352591 10.1186/s12909-018-1347-3 PMC6199751

[38] Chang B-Y, Chang C-Y, Hwang G-H, Kuo F-R. A situation-based flipped classroom to improving nursing staff performance in advanced cardiac life support training course. Interact Learn Environ. 2019;27:1062–74.

[39] Subramaniam T, Valuyeetham PS, Siang TJ. Students’ feedback on effectiveness of combined flipped classroom and high fidelity simulated teaching on airway and ventilation during accident and emergency posting. Educ Med J. 2018;10:5–13.

[40] Moll-Khosrawi P, Zöllner C, Cencin N, Schulte-Uentrop L. Flipped learning enhances non-technical skill performance in simulation-based education: a randomised controlled trial. BMC Med Educ. 2021;21:353. 34158030 10.1186/s12909-021-02766-w PMC8220780

[41] McLean S, Attardi SM. Sage or guide? Student perceptions of the role of the instructor in a flipped classroom. Act Learn High Educ. 2018;24:49–61.

[42] Zheng B, Zhang Y. Self-regulated learning: the effect on medical student learning outcomes in a flipped classroom environment. BMC Med Educ. 2020;20:100. 32234040 10.1186/s12909-020-02023-6 PMC7110809

[43] Wong WJ, Lee SWH, White PJ, Efendie B, Lee RFS. Perspectives on opportunities and challenges in a predominantly flipped classroom-based pharmacy curriculum: a qualitative study. Curr Pharm Teach Learn. 2023;15:242–51. 37055316 10.1016/j.cptl.2023.03.004

[44] Narciss S. Feedback strategies for interactive learning tasks. In: Spector JM, Merrill MD, van Merrenboer JJG, Driscoll MP, editors. Handbook of research on educational communications and technology. London: Routledge; 2008.p125–143.

[45] Sourg HAA, Satti S, Ahmed N, Ahmed ABM. Impact of flipped classroom model in increasing the achievement for medical students. BMC Med Educ. 2023;23:287. 37106403 10.1186/s12909-023-04276-3 PMC10142149

[46] Meyer DE, Schvaneveldt RW. Facilitation in recognizing pairs of words: evidence of a dependence between retrieval operations. J Exp Psychol. 1971;90:227. 5134329 10.1037/h0031564

