# Supplementary material for: A flipped classroom improves medical students’ skills how to manage medical emergencies—an intervention-control study
Source: BMC Med Educ. 2025 Oct 7;25:1362. doi: 10.1186/s12909-025-07898-x (PMC12502129; doi:10.1186/s12909-025-07898-x)
Supplement: Supplementary file 1 — Supplementary Material 1. [file 12909_2025_7898_MOESM1_ESM.docx]

**Supplementary File 1 – Checklists in German**

**Station Sepsis**

TN-Nummer: _______________________ KW: _______________________

Prüfende: _______________________

| **Durchführung** | **Nein** | **Ja** |
| --- | --- | --- |
| **Allgemeines** | | |
| Eigensicherung (Handschuhe) |  |  |
| Ansprechen/ Schmerzreiz |  |  |
| Notruf/ Stationsalarm |  |  |
| Einbinden Helfer |  |  |
| ABCDE Schema eingehalten |  |  |
| Korrekter Umgang mit dem Helfer |  |  |
| Setting optimiert |  |  |

| **Durchführung** | | **Nicht durchgeführt**  **0 Punkte** | | **Nur teilweise und/oder inkorrekt durchgeführt**  **1 Punkt** | | **Vollständig und korrekt durchgeführt**  **2 Punkte** | |
| --- | --- | --- | --- | --- | --- | --- | --- |
| **A – Airway** | | | | | | | |
| Prüfung, ob Atemwege frei sind | |  | |  | |  | |
| **B – Breathing** | | | | | | | |
| Kontrolle der Atmung  (*10s hören-sehen-fühlen)* | |  | |  | |  | |
| Lunge auskultieren (*unauffällig*) | |  | |  | |  | |
| AF ausgezählt (*Tachy = 1 Punkt, 22/min = 2 Punkte*) | |  | |  | |  | |
| SpO2 erheben  (*SpO2 = 80%)* | |  | |  | |  | |
| **C - Circulation** | | | | | | | |
| ReKap Zeit prüfen  (*3s, Extremitäten sind kalt*) | |  | |  | |  | |
| RR prüfen  (*95/60)* | |  | |  | |  | |
| Puls prüfen  (*110/min*) | |  | |  | |  | |
| EKG interpretieren:  HF und Rhythmus erkennen  (*Sinustachykardie*), *bei 1 = 1 Punkt, bei 2 = 2 Punkte* | |  | |  | |  | |
| Weiteren großlumigen i.v.-Zugang etablieren (*1 Punkt*) und Infusion anhängen (*2 Punkte*) | |  | |  | |  | |
| **D – Disability** | | | | | | | |
| Pupillen beurteilen  (*mittelweit, isocor*) | |  | |  | |  | |
| Vigilanzminderung benennen | |  | |  | |  | |
| Temperatur erheben  (*39,4°)* | |  | |  | |  | |
| BZ messen  (*120 mg/dl*) | |  | |  | |  | |
| **E - Exposure/Environment** | | | | | | | |
| Patient entkleiden | |  | |  | |  | |
| Body-Check: Infektionsquelle Katheter erkannt | |  | |  | |  | |
| **Anamnese** (mit Hilfe der Tochter) | | | | | | | |
| Symptome  (*Leitsymptom = teils, OPQRST min. 3/6 = vollständig.*) | |  | |  | |  | |
| Allergien | |  | |  | |  | |
| Medikamente | |  | |  | |  | |
| Patientenvorgeschichte | |  | |  | |  | |
| Letzte… | |  | |  | |  | |
| Ereignis | |  | |  | |  | |
| Risikofaktoren | |  | |  | |  | |
| **Weiteres Patientenmanagement**  *Prüfer unterbricht nach 7 min und erfragt weiteres Vorgehen bei Studierenden* | | | | | | | |
| Volumengabe anordnen | |  | |  | |  | |
| Körperliche Untersuchung | |  | |  | |  | |
| Blutkultur abnehmen (*anfordern = teils, ausdrücklich vor Antibiose = vollständig*) | |  | |  | |  | |
| Breitbandantibiose anordnen  *1 Punkt, wenn erwähnt; 2 Punkte, wenn Standard-AB genannt wird, z.B. Ceftriaxon, Pip-Taz o.Ä.* | |  | |  | |  | |
| Verlegung auf ICU/IMC | |  | |  | |  | |
| **Struktur: Inwiefern entsprach der Ablauf dem gelehrten Algorithmus?** | | | | | | | |
| ABCDE in korrekter Reihenfolge durchgeführt und Fremdanamnese strukturiert erhoben. | | | | | | | |
| Kaum bis gar nicht strukturiert |  | | Grundstruktur vorhanden, aber mit Auslassungen und Einschüben | |  | | Führt Maßnahmen wie in der Checkliste durch |
| 1 | 2 | | 3 | | 4 | | 5 |

**Station BLS AED**

TN-Nummer: _______________________ KW: _______________________

Prüfende: _______________________

| **Durchführung** | **Nein**  **0 Punkte** | **Ja**  **1 Punkt** |
| --- | --- | --- |
| **Allgemeines** | | |
| Eigensicherung (Handschuhe) |  |  |
| Ansprechen/ Schmerzreiz |  |  |
| Notruf/ Stationsalarm |  |  |
| Einbinden Helfer |  |  |
| ABCDE Schema eingehalten |  |  |
| Korrekter Umgang mit dem Helfer |  |  |
| Setting optimiert |  |  |

| **Durchführung** | **Nicht durchgeführt**  **0 Punkte** | **Nur teilweise und/oder inkorrekt durchgeführt**  **1 Punkt** | **Vollständig und korrekt durchgeführt**  **2 Punkte** |
| --- | --- | --- | --- |
| **A – Airway** | | | |
| Atemwege prüfen |  |  |  |
| Kopf überstrecken |  |  |  |
| Inspektion Mundraum |  |  |  |
| **B – Breathing** | | | |
| Kontrolle  (*10s sehen, hören, fühlen*) |  |  |  |
| Reanimationsindikation stellen  (*Keine Atmung*) |  |  |  |
| Effektive Beutel-Maske-Beatmung (*1 Punkt, wenn beatmet, 2, wenn Beatmung suffizient*) |  |  |  |
| **C - Circulation (Beurteilung wenn TN selbst drückt, nicht die Assistenz)** | | | |
| CPR beginnen |  |  |  |
| Drucktiefe: 5-6 cm |  |  |  |
| Druckfrequenz 100-120/min |  |  |  |
| Zyklus: 30:2 CPR/Beatmung |  |  |  |
| Entlastung beachtet |  |  |  |
| **D- Defibrillation** | | | |
| Korrektes Handling des AED  (*Anschalten, Aufkleben, Anschließen, Analyse*) |  |  |  |
| Korrekte Kommandos  (*Weg vom Patienten; Achtung: Schock*) |  |  |  |
| Kontrolle vor Schockabgabe |  |  |  |
| Abgabe des Schocks |  |  |  |
| Zügig weiter mit CPR  (*hands-off-Zeit < 10s*) |  |  |  |
| Korrekte Weiterführung des 30:2 Algorithmus |  |  |  |
| **Weiteres Patientenmanagement**  *Prüfer unterbricht nach 7 min und erfragt weiteres Vorgehen bei Studierenden* | | | |
| Nach erfolgreicher Reanimation (= Patient hat Kreislauf, wird angesagt) | | | |
| Atmung überprüfen |  |  |  |
| Stabile Seitenlage (*Ansagen reicht*) |  |  |  |
| Situation  (*Name, Alter, VD*) |  |  |  |
| Aktuelle Anamnese  (*Symptome, Dauer*) |  |  |  |
| Fakten  (*Befunde, Maßnahmen*) |  |  |  |
| Entwicklung  (*Verlauf +/-*) |  |  |  |

| **Struktur: Inwiefern entsprach der Ablauf dem gelehrten Algorithmus?** | | | | |
| --- | --- | --- | --- | --- |
| ABC in korrekter Reihenfolge durchgeführt und Maßnahmen richtig priorisiert  (1. CPR, 2. Defibrillation) | | | | |
| Kaum bis gar nicht strukturiert |  | Grundstruktur vorhanden, aber mit Auslassungen und Einschüben |  | Führt Maßnahmen wie in der Checkliste durch |
| 1 | 2 | 3 | 4 | 5 |

**Station VF**

TN-Nummer: _______________________ KW: _______________________

Prüfende: _______________________

| **Durchführung** | **Nein**  **0 Punkte** | **Ja**  **1 Punkte** |
| --- | --- | --- |
| **Allgemeines** | | |
| Eigensicherung (Handschuhe) |  |  |
| Ansprechen/ Schmerzreiz |  |  |
| Notruf/ Stationsalarm |  |  |
| Einbinden Helfer |  |  |
| ABCDE Schema eingehalten |  |  |
| Korrekter Umgang mit dem Helfer |  |  |
| Setting optimiert |  |  |

| **Durchführung** | **Nicht durchgeführt**  **0 Punkte** | **Nur teilweise und/oder inkorrekt durchgeführt**  **1 Punkt** | **Vollständig und korrekt durchgeführt**  **2 Punkte** |
| --- | --- | --- | --- |
| **A – Airway** | | | |
| Kopf überstrecken |  |  |  |
| Inspektion Mundraum |  |  |  |
| Prüfung, ob Atemwege frei sind |  |  |  |
| **B - Breathing** | | | |
| Kontrolle der Atmung, 10s  (Sehen, Hören, Fühlen)  *(Geprüft = teils, alle Kriterien erfüllt = vollständig)* |  |  |  |
| Reanimation wird eingeleitet  (*da keine Atmung feststellbar*) |  |  |  |
| Beutel-Masken-Beatmung (*in 30:2 Rhythmus*) |  |  |  |
| Ggf. erweitertes Atemwegsmanagement (*muss bloß erwähnt werden*) |  |  |  |
| **C - Circulation** | | | |
| CPR wird begonnen (*30:2*) |  |  |  |
| Rea-Board/ harte Unterlage wird verwendet |  |  |  |
| Setting optimieren: Kopfteil flach, Bett/Liege weg von der Wand, Material sinnvoll arrangiert, Helfer eingebunden  (*>2 = 2 Punkte*) |  |  |  |
| **C- Unterpunkt Defi** | | | |
| Defi wird angebracht (*2 Punkte, wenn Position korrekt*) |  |  |  |
| Rhythmusanalyse mit Erkennen des angezeigten Rhythmus (*Analyse = 1 Punkt, VF erkennen = 2 Punkte*) |  |  |  |
| Auf Sicherheit wird geachtet: *Kommando: „Weg vom Patienten“* |  |  |  |
| Laut und deutlich das Kommando: *„Achtung, Schock!“* |  |  |  |
| Fortführung CPR nach Schock (*2 Pkt wenn hands-off-time < 10s*) |  |  |  |
| i.V. Zugang (*1 Punkt*) und Infusion werden etabliert (*2 Punkte*) |  |  |  |
| **Weiteres Patientenmanagement (Medikamente, Airway)**  *Prüfer unterbricht nach 3. Schock / 7 min und erfragt weiteres Vorgehen bei Studierenden* | | | |
| Erneute Rhythmusanalyse nach 2min *(Erkennen VF persistiert)* |  |  |  |
| 2. Schockabgabe (*mit Kommandos*) |  |  |  |
| Fortführung CPR nach Schock (*2 Pkt wenn hands-off-time < 10s*) |  |  |  |
| Erneute Rhythmusanalyse nach 2min *(Erkennen VF persistiert)* |  |  |  |
| 3. Schockabgabe  *(mit Kommandos)* |  |  |  |
| Erweitertes Airway-Management |  |  |  |
| Adrenalin verabreicht (*alle 3-5 Min, 1 mg auf 9 NaCl*)  (*1 Punkt, wenn angeordnet, 2 Punkte, wenn Dosierung korrekt*) |  |  |  |
| Amiodaron verabreicht (*300 mg/150mg/(150mg) auf G5%*)  (*1 Punkt, wenn angeordnet, 2 Punkte, wenn Dosierung korrekt*) |  |  |  |

| **Struktur: Inwiefern entsprach der Ablauf dem gelehrten Algorithmus?** | | | | |
| --- | --- | --- | --- | --- |
| ABC in korrekter Reihenfolge durchgeführt und Maßnahmen richtig priorisiert  (1. CPR, 2. Defibrillation, 3. Airway/Medikamente) | | | | |
| Kaum bis gar nicht strukturiert |  | Grundstruktur vorhanden, aber mit Auslassungen und Einschüben |  | Führt Maßnahmen wie in der Checkliste durch |
| 1 | 2 | 3 | 4 | 5 |

**Station Infarkt**

TN-Nummer: _______________________ KW: ____________________

Prüfende: _______________________

| **Durchführung** | **Nein**  **0 Punkte** | **Ja**  **1 Punkt** |
| --- | --- | --- |
| **Allgemeines** | | |
| Eigensicherung (Handschuhe) |  |  |
| Ansprechen/ Schmerzreiz |  |  |
| Notruf/ Stationsalarm |  |  |
| Einbinden Helfer |  |  |
| ABCDE Schema eingehalten |  |  |
| Korrekter Umgang mit dem Helfer |  |  |
| Setting optimiert |  |  |

| **Durchführung** | **Nicht durchgeführt**  **0 Punkte** | **Nur teilweise und/oder inkorrekt durchgeführt**  **1 Punkt** | **Vollständig und korrekt durchgeführt**  **2 Punkte** |
| --- | --- | --- | --- |
| **A - Airway** | | | |
| Prüfung, ob Atemwege frei sind |  |  |  |
| **B – Breathing** | | | |
| Auskultation |  |  |  |
| AF auszählen (*Tachy = 1 Punkt, 22/min = Punkt*) |  |  |  |
| Pulsoxymeter anschließen  (*SpO2 95%*) |  |  |  |
| **C - Circulation** | | | |
| RR-Messung  *(160/100 mmHg = teils, RR re-evaluiert nach Nitrogabe = vollständig)* |  |  |  |
| Puls tasten  (*Tachy = 1 Punkt, 140/min = 2 Punkte*) |  |  |  |
| IV-Zugang  *(Zugang anordnen = teils, Infusion anschließen = vollständig)* |  |  |  |
| ReKap (*2s*) |  |  |  |
| **EKG**  (4-Kanal) |  |  |  |
| **STEMI erkannt** |  |  |  |
| **12-Kanal nachfordern** |  |  |  |
| **D - Disability** | | | |
| Schwindel, Übelkeit festgestellt |  |  |  |
| BZ-Messung |  |  |  |
| Pupillen untersucht |  |  |  |
| **E - Environment/Exposure** | | | |
| Patient entkleidet/ Oberkörper frei gemacht |  |  |  |
| Temperatur messen |  |  |  |
| **Anamnese** | | | |
| **Symptom** Leitsymptom (*Thoraxschmerz*) |  |  |  |
| **Onset**  Beginn der Symptome (*plötzlich vor 15 min*) |  |  |  |
| **Provocation** Verbesserung/Verschlechterung (*durchgehend*) |  |  |  |
| **Quality**  Schmerzqualität (*schneidend*) |  |  |  |
| **Radiation**  Ausstrahlung (*linke Schulter*) |  |  |  |
| **Severity**  Intensität (*7-8*) |  |  |  |
| **Time**  Änderung im Verlauf? In Ruhe/ Bei Belastung?  *(konstant, in Ruhe)* |  |  |  |
| **Allergien** |  |  |  |
| **Medikamente**  *(1 Punkt, wenn allgemein gefragt;*  *bei konkreter Frage nach PDE-5-Hemmern = 2 Punkte)* |  |  |  |
| **Patientenvorgeschichte** |  |  |  |
| **Letzte Mahlzeit** |  |  |  |
| **Event**  Vorkommnis, das das Leitsymptom ausgelöst haben könnte? |  |  |  |
| **Risikofaktoren**  (*Rauchen, Cholesterin*) |  |  |  |
| **Therapie** | | | |
| Morphin 3-5 mg i.v. (*Gabe = teils, korrekte Dosierung = vollständig*) |  |  |  |
| O2-Gabe erfolgt |  |  |  |
| Nitro (*Gabe = teils, RR berücksichtigen = vollständig*) |  |  |  |
| ASS 500 mg i.v. (*Gabe = teils, Dosierung = vollständig*) |  |  |  |
| Heparin 5000 I.E. *(Gabe = teils, Dosierung = vollständig)* |  |  |  |
| Antiemetikum bei Morphin-Gabe (*Vomex i.v.*) |  |  |  |
| **Weiteres Patientenmanagement (Medikamente, Airway)**  *Prüfer unterbricht nach 7 min und erfragt weiteres Vorgehen bei Studierenden* | | | |
| Transport in Klinik (*Ansage = teils, mit Katheterlabor = vollständig*) |  |  |  |
| Oberkörperhochlagerung (*1* *Punkt geben wenn keine Flachlagerung stattfindet*) |  |  |  |

| **Struktur: Inwiefern entsprach der Ablauf dem gelehrten Algorithmus?** | | | | |
| --- | --- | --- | --- | --- |
| ABCD in korrekter Reihenfolge durchgeführt und Anamnese strukturiert geführt. | | | | |
| Kaum bis gar nicht strukturiert |  | Grundstruktur vorhanden, aber mit Auslassungen und Einschüben |  | Führt Maßnahmen wie in der Checkliste durch |
| 1 | 2 | 3 | 4 | 5 |

**Station Asthma**

TN-Nummer: _______________________ KW: ____________________

Prüfende: _______________________

| **Durchführung** | **Nein**  **0 Punkte** | **Ja**  **1 Punkt** |
| --- | --- | --- |
| **Allgemeines** | | |
| Eigensicherung (Handschuhe) |  |  |
| Ansprechen/ Schmerzreiz |  |  |
| Notruf/ Stationsalarm |  |  |
| Einbinden Helfer |  |  |
| ABCDE Schema eingehalten |  |  |
| Korrekter Umgang mit dem Helfer |  |  |
| Setting optimiert |  |  |

| **Durchführung** | **Nicht durchgeführt**  **0 Punkte** | **Nur teilweise und/oder inkorrekt durchgeführt**  **1 Punkt** | **Vollständig und korrekt durchgeführt**  **2 Punkte** |
| --- | --- | --- | --- |
| **A - Airway** | | | |
| Prüfung, ob Atemwege frei sind |  |  |  |
| Atemgeräusch erkennen (exsp. Stridor) |  |  |  |
| **B – Breathing** | | | |
| Tachypnoe feststellen |  |  |  |
| AF auszählen (*Tachy = 1 Punkt, 25/min = Punkt*) |  |  |  |
| Pulsoxymeter anschließen  (*SpO2 90%*) |  |  |  |
| O2 Gabe |  |  |  |
| Auskultation (1 = teils, Giemen und Brummen identifiziert: = vollständig 2) |  |  |  |
| **C - Circulation** | | | |
| RR-Messung  *anfordern* |  |  |  |
| Puls tasten  (*Tachy = 1 Punkt, 140/min = 2 Punkte*) |  |  |  |
| IV-Zugang  *(Zugang anordnen = teils, Infusion anschließen = vollständig)* |  |  |  |
| ReKap (1-*2s*) |  |  |  |
| **EKG**  (4-Kanal) anfordern = 1 Punkt; 2 = richtig beurteilt |  |  |  |
| iv-Zugang + Infusion (je 1 Punkt) |  |  |  |
| **D - Disability** | | | |
| Erkennt, dass kein D-Problem vorliegt |  |  |  |
| **E - Environment/Exposure** | | | |
| Patient entkleidet/ Oberkörper frei gemacht |  |  |  |
| **Anamnese** | | | |
| **Symptome** benannt= teils Beginn/Verlauf/Auslöser...(mind. 2)= vollständig |  |  |  |
| **Allergien** |  |  |  |
| **Medikamente**  *(1 Punkt, wenn allgemein gefragt;*  *bei konkreter Frage nach PDE-5-Hemmern = 2 Punkte)* |  |  |  |
| **Patientenvorgeschichte** |  |  |  |
| **Letzte Mahlzeit** |  |  |  |
| **Event**  Vorkommnis, das das Leitsymptom ausgelöst haben könnte? |  |  |  |
| **Risikofaktoren**  (*Rauchen, Cholesterin*) |  |  |  |
| **Weiteres Patientenmanagement (Medikamente, Airway)**  *Prüfer unterbricht nach 7 min und erfragt weiteres Vorgehen bei Studierenden* | | | |
| Verdachtsdiagnose |  |  |  |
| Nach eigenem Asthmaspray gefragt(bereits erfolglos benutzt) |  |  |  |
| Atrovent 0,5 mg über Verneblermaske |  |  |  |
| Prednisolon 50-100 mg (angedacht= teils, korrekte Dosierung verabreicht= vollständig) |  |  |  |
| Mitnahme in die Klinik |  |  |  |

| **Struktur: Inwiefern entsprach der Ablauf dem gelehrten Algorithmus?** | | | | |
| --- | --- | --- | --- | --- |
| ABCD in korrekter Reihenfolge durchgeführt und Anamnese strukturiert geführt. | | | | |
| Kaum bis gar nicht strukturiert |  | Grundstruktur vorhanden, aber mit Auslassungen und Einschüben |  | Führt Maßnahmen wie in der Checkliste durch |
| 1 | 2 | 3 | 4 | 5 |

**Station Stroke**

TN-Nummer: _______________________ KW: ____________________

Prüfende: _______________________

| **Durchführung** | **Nein**  **0 Punkte** | **Ja**  **1 Punkt** |
| --- | --- | --- |
| **Allgemeines** | | |
| Eigensicherung (Handschuhe) |  |  |
| Ansprechen/ Schmerzreiz |  |  |
| Einbinden Helfer |  |  |
| Korrekter Umgang mit dem Helfer*innen |  |  |
| Setting optimiert |  |  |

| **Durchführung** | **Nicht durchgeführt**  **0 Punkte** | **Nur teilweise und/oder inkorrekt durchgeführt**  **1 Punkt** | **Vollständig und korrekt durchgeführt**  **2 Punkte** |
| --- | --- | --- | --- |
| **A - Airway** | | | |
| Prüfung, ob Atemwege frei sind |  |  |  |
| **B – Breathing** | | | |
| AF auszählen (*Tachy = 1 Punkt, 16/min = 2 Punkte*) |  |  |  |
| Anordnung Pulsoxymeter anschließen  (*SpO2 90%*) |  |  |  |
| Auskultation |  |  |  |
| **C - Circulation** | | | |
| RR-Messung  *anfordern* |  |  |  |
| Puls tasten  (*Tachy = 1 Punkt, 140/min = 2 Punkte*) |  |  |  |
| IV-Zugang  *(Zugang anordnen = teils, Infusion anschließen = vollständig)* |  |  |  |
| ReKap (1-*2s*) |  |  |  |
| **EKG**  (4-Kanal) anfordern = 1 Punkt; 2 = richtig beurteilt |  |  |  |
| iv-Zugang + Infusion (je 1 Punkt) |  |  |  |
| **D - Disability** | | | |
| Pupillen beurteilen (mittelweit, rund, jeweils prompte direkte/indirekte LR) |  |  |  |
| FACE Untersuchung der mimischen Muskulatur |  |  |  |
| ARMS Untersuchung der Motorik |  |  |  |
| SPEECH Untersuchung der Sprache |  |  |  |
| TimeVD Apoplex, Time ist brain, schnell in die Stroke Unit |  |  |  |
| Fragen, ob paretische Symptome schon bestanden |  |  |  |
| DD: BZ Messung (120mg/dl) |  |  |  |
| **E - Environment/Exposure** | | | |
| Patient entkleidet/ Oberkörper frei gemacht |  |  |  |
| DD: Temp messen |  |  |  |
| **Anamnese** | | | |
| **Symptome** benannt= teils Beginn/Verlauf/Auslöser...(mind. 2)= vollständig |  |  |  |
| **Allergien** |  |  |  |
| **Medikamente**  *(1 Punkt, wenn allgemein gefragt;*  *bei konkreter Frage nach PDE-5-Hemmern = 2 Punkte)* |  |  |  |
| **Patientenvorgeschichte** |  |  |  |
| **Letzte Mahlzeit** |  |  |  |
| **Event**  Vorkommnis, das das Leitsymptom ausgelöst haben könnte? |  |  |  |
| **Risikofaktoren**  (*Rauchen, Cholesterin*) |  |  |  |
| **Weiteres Patientenmanagement (Medikamente, Airway)**  *Prüfer unterbricht nach 7 min und erfragt weiteres Vorgehen bei Studierenden* | | | |
| Anordnen eines schnellen Transports: Transport= teils, Zusatz KH mit Stroke-Unit= vollständig |  |  |  |
| Vorgehen: cCT Ausschluss Blutung |  |  |  |
| Lysetherapie (Zeitfenster 4,5h) |  |  |  |

| **Struktur: Inwiefern entsprach der Ablauf dem gelehrten Algorithmus?** | | | | |
| --- | --- | --- | --- | --- |
| ABCD in korrekter Reihenfolge durchgeführt und Anamnese strukturiert geführt. | | | | |
| Kaum bis gar nicht strukturiert |  | Grundstruktur vorhanden, aber mit Auslassungen und Einschüben |  | Führt Maßnahmen wie in der Checkliste durch |
| 1 | 2 | 3 | 4 | 5 |
